# Intraoperative Neuromonitoring Changes During MIS-TLIF Associated with Postoperative Foot Drop

**DOI:** 10.3390/jcm15155795

**Published:** 2026-07-24

**Authors:** Michael C. Oblich, James G. Lyman, James M. Mossner, Tyler Koski, Najib El Tecle, Nader Dahdaleh, Kevin Swong

**Affiliations:** 1 Feinberg School of Medicine, Northwestern University, Chicago, IL 60611, USA; james.lymaniii@northwestern.edu; 2 Department of Neurological Surgery, Northwestern University, Chicago, IL 60611, USA; james.mossner@nm.org (J.M.M.); tyler.koski@nm.org (T.K.); najib.eltecle@nm.org (N.E.T.); nader.dahdaleh@northwestern.edu (N.D.); kevin.swong@nm.org (K.S.)

**Keywords:** foot drop, intraoperative neuromonitoring, lumbar fusion, minimally invasive spine surgery, MIS-TLIF

## Abstract

**Background/Objectives:** Intraoperative neuromonitoring (IOM) is commonly used in instrumented spine surgery; however, its utility is often debated. We sought to determine if IOM changes were independently associated with specific postoperative outcomes in minimally invasive transforaminal lumbar interbody fusion (MIS-TLIF). **Methods:** We performed a retrospective analysis of patients who underwent MIS-TLIF with IOM at a single institution. Collected data included demographics, comorbidities, surgical history, operative indications, operative spinal levels, IOM findings, and postoperative outcomes. Single and multivariate logarithmic regression analyses were performed to assess the correlation of each preoperative variable with IOM alerts, as well as the association between IOM alerts and postoperative outcomes. **Results:** A total of 439 patients were identified, 21 of whom experienced an IOM alert. No preoperative variables were significantly associated with IOM alerts. However, the presence of an IOM alert significantly increased the log odds of postoperative foot drop (1.93–6.55, *p* < 0.001), for which there was also a high specificity (95.9%) and negative predictive value (99.8%). However, likely due to low event rates, IOM had a low negative predictive value (16.7%). Further multivariate analysis also identified an IOM alert as a significant predictor of postoperative neurological deficits and chronic complications, although these pooled categories included foot drop. When IOM subtypes were investigated, both the SSEP and MEP significantly increased the log odds of postoperative foot drop (*p* < 0.001). **Conclusions:** An IOM alert during MIS-TLIF may be associated with chronic complications and postoperative neurological deficits, particularly foot drop. These findings highlight IOM’s potential use as an effective intraoperative screening tool to rule out iatrogenic foot drop but also its limited value to independently predict rare postoperative complications.

## 1. Introduction

Intraoperative monitoring (IOM) is a perioperative technique used to assess the functional integrity of the nervous system via continuous monitoring of electrophysiological signals from the brain, spinal cord, and peripheral nerves [1]. One previous retrospective study estimated that IOM was utilized in 15.69% of 15,326 total lumbar fusions [2]. Minimally invasive transforaminal lumbar interbody fusion (MIS-TLIF) is one of the most commonly performed lumbar fusion procedures and frequently uses IOM. First introduced by Foley et al. in 2003, the MIS-TLIF approach allows for decompression and interbody fusion via small, bilateral incisions on the dorsal side [3].

The use of IOM with MIS-TLIF aims to minimize postoperative complications resulting from iatrogenic damage to the spinal cord and nerve roots. Previous studies have indicated that IOM is sensitive and specific for detecting intraoperative injury, providing surgeons a chance to reduce potential harm and anticipate postoperative complications [4,5]. However, there are conflicting claims on whether patient outcomes in spine surgery are meaningfully improved by the use of IOM [6,7]. Thus, the ultimate value of IOM in MIS-TLIF is a frequently debated topic, especially given the high costs associated with its utilization [8,9].

It has been proposed that the potential benefit of IOM comes from an experienced surgeon’s interpretation of alerts and the corrective measures taken thereafter [10]. If this is truly the case, then it is imperative that surgeons know what complications to be vigilant to in order to proactively address them. Previous studies have shown that IOM alerts can be useful in predicting postoperative neurological deficits in general, but the literature describing the specific complications predicted by IOM is limited [11,12,13]. In this study, we sought to characterize the association between IOM changes and postoperative neurologic outcomes in patients undergoing MIS-TLIF.

## 2. Materials and Methods

### 2.1. Study Design, Setting, and Participants

We performed a retrospective analysis of a cohort of 439 patients who underwent MIS-TLIF at a single institution between January 2010 and May 2021. All patients were identified using the institution’s electronic data warehouse (EDW). This study was approved by our Institutional Review Board (IRB No. STU00214662), conducted in accordance with Health Insurance Portability and Accountability Act (HIPAA) guidelines, and reported in accordance with Strengthening the Reporting of Observational Studies in Epidemiology (STROBE) guidelines. Inclusion criteria for this study were patients over the age of 18 who underwent MIS-TLIF at our institution within the previously specified time period. A total of 573 patients were initially identified via the electronic data warehouse. Patients were then excluded during the initial screening of EDW data if they underwent a procedure other than MIS-TLIF (*n* = 76), had fusion of four or more spinal levels (*n* = 2), had trauma (*n* = 1) or osteomyelitis (*n* = 2), were under the age of 18 (*n* = 2), or were missing significant follow-up data (*n* = 6). Of the 486 patients remaining, 47 were excluded from our analysis due to a lack of a baseline IOM recording, leaving 439 for inclusion in our analysis. All included patients underwent MIS-TLIF with IOM utilizing a standardized intraoperative monitoring anesthesia protocol, limiting volatile anesthetic use to a ≤0.5 minimum alveolar concentration (MAC) and utilizing total intravenous anesthesia (TIVA) as indicated.

### 2.2. Data Collection

Collected demographic variables included patient age at the time of surgery, sex, body mass index (BMI), smoking status, and drug use. Clinical variables included history of previous spine surgery, history of deep vein thrombosis (DVT) or pulmonary embolism (PE), preoperative American Society of Anesthesiologists (ASA) Physical Status Classification System score, indications for surgery, and presence of an IOM alert. IOM alerts were identified by monitoring physicians based on standardized, modality-dependent criteria. For the somatosensory evoked potential (SSEP), the alert threshold was a 50% decrease in amplitude with an associated 10% increase in latency in comparison to the patient’s baseline values. A motor evoked potential (MEP) alert was triggered by a reduction of more than 50% of the baseline amplitude. Electromyography (EMG) was free running and alerted with any positive changes. Outcome variables included both intraoperative and postoperative complications, length and course of hospital stay, and Karnofsky score preoperatively, at discharge, and at follow-up. Deidentified data is available upon request.

### 2.3. Statistical Analysis

Statistical analysis was performed using R (version 4.1.1, R Foundation for Statistical Computing, Vienna, Austria). Log odds, 95% confidence intervals, and the associated *p*-values were calculated using univariate logistic regression for categorical variables and univariate linear regression for numerical variables. Multivariate logistic regression was performed when possible. A *p*-value < 0.05 was considered statistically significant for all analyses. 

### 2.4. Artificial Intelligence Use

During the preparation of this manuscript, the authors used ChatGPT, Version 5.2, for the sole purposes of proofreading and formatting. All use of AI was compliant with journal policies. The authors have reviewed and edited the output and take full responsibility for the content of this publication.

## 3. Results

An IOM alert was identified in 21 of 439 (4.8%) patients included in our analysis. The overall demographics and prevalence of IOM alerts in each demographic and surgical indication group are shown in Table 1 and Table 2. There were no significant demographic differences identified between any of the groups. Of note, many patients presented with several different indications for MIS-TLIF, which accounts for the greater than 439 patients presented in Table 2.

**Figure 1:**
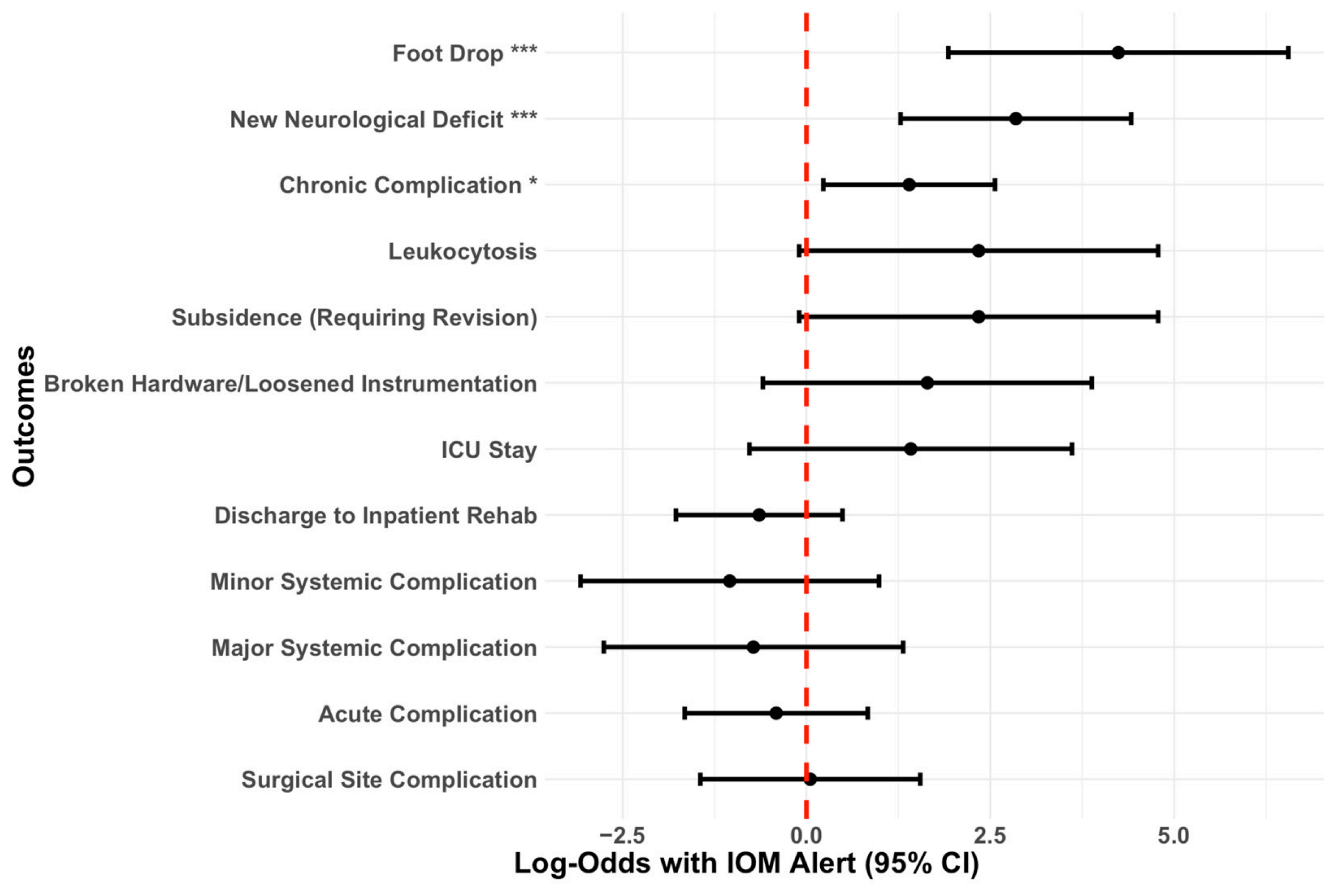
**IOM alert predicting postoperative outcomes**. The presence of an IOM alert was strongly associated with postoperative foot drop, increasing the log odds of this complication more than 4 times. IOM alerts also significantly increased the log odds of postoperative neurological deficits and chronic complications lasting longer than 6 months, though it should be noted that foot drop was included in both composite categories. Outcomes with *p* > 0.95 were excluded from the figure for clarity. (*** *p* < 0.001, * *p* < 0.05).

**Figure 2:**
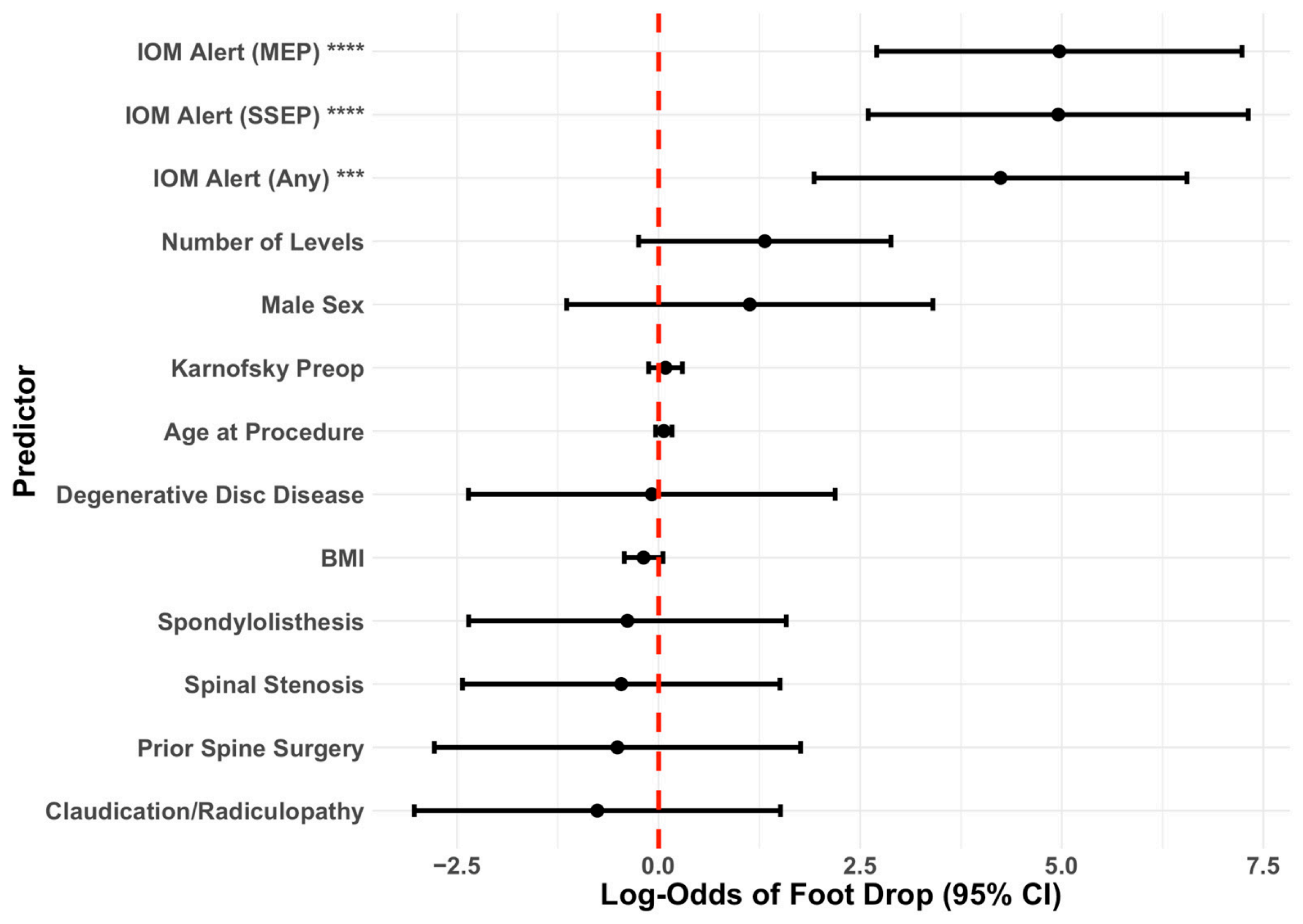
**Univariate predictors of foot drop.** Univariate logistic regression identified IOM alerts as the only significant predictor of postoperative foot drop out of the identified predictor variables. IOM alerts involving changes in the SSEP and/or MEP had the highest association (outcomes with *p* > 0.95 were excluded for clarity). (*** *p* < 0.001, **** *p* < 10^−4^).

In our cohort, an IOM alert was found to be strongly associated with postoperative foot drop (log odds 4.24; 95% CI: 1.93–6.55; *p* = 0.0003) (Figure 1). Further univariate analysis identified an IOM alert as the only significant predictor of postoperative foot drop out of the previously identified demographic variables and surgical indications. The number of spinal levels involved in the procedure was the only other predictor variable approaching significance. When IOM subtypes were investigated, both SSEP (*p* = 3.78 × 10^−5^) and MEP (*p* = 1.71 × 10^−5^) changes significantly increased the log odds of postoperative foot drop (Figure 2). The IOM alert demonstrated a high specificity (95.9%) and negative predictive value (99.8%) for postoperative foot drop, along with moderate sensitivity (75%). However, the positive predictive value was only 14.3%, likely as a result of the low prevalence (0.9%) of foot drop in our cohort.

**Figure 3:**
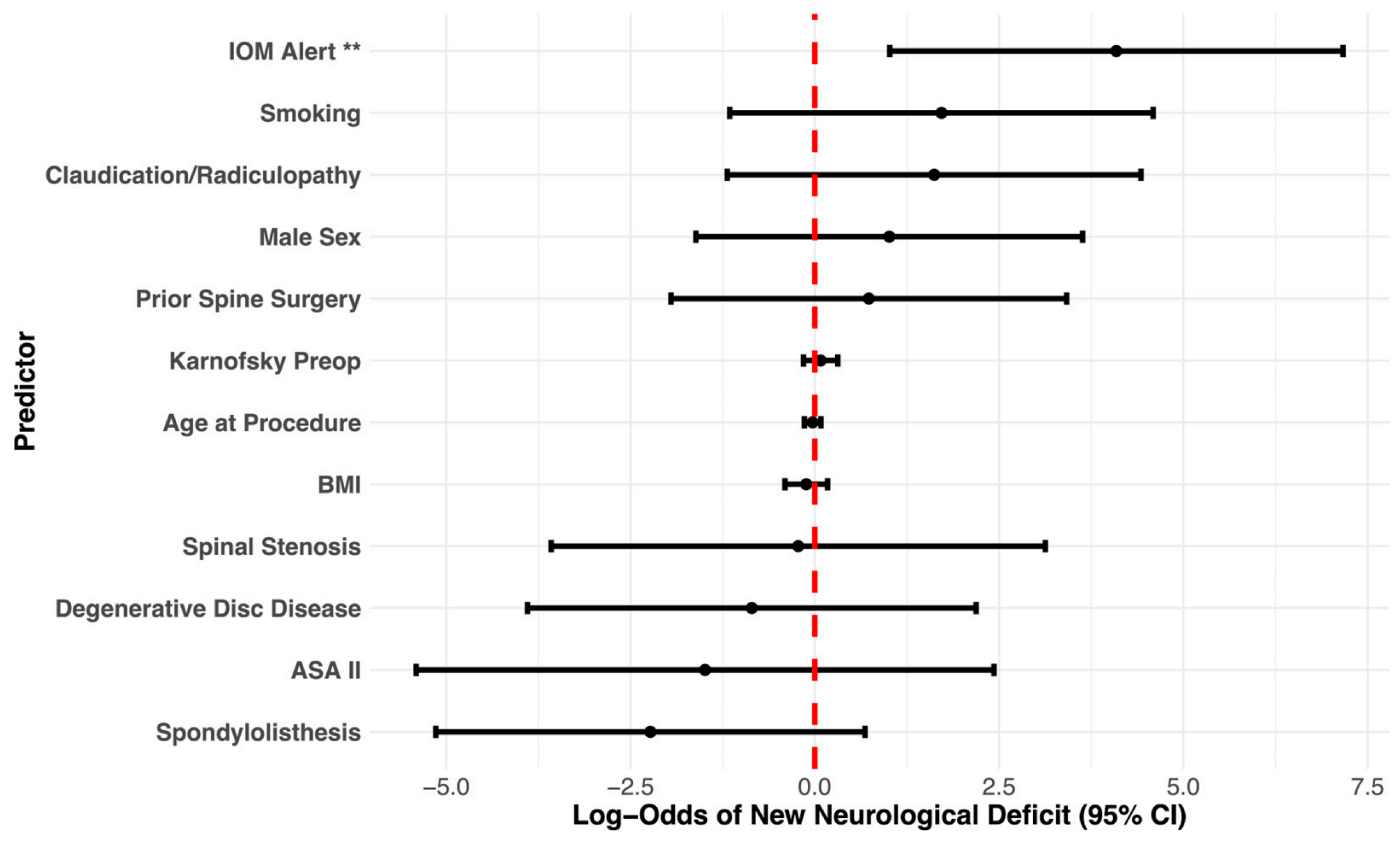
**Multivariate predictors of new neurological deficit.** Of the 22 variables included in our multivariate model, an IOM alert was the only significant predictor of new postoperative neurological deficit, increasing the log odds of a patient presenting with one of more of postoperative foot drop, nerve root injury (excluding foot drop), or cauda equina syndrome by more than 4 times. Predictors with *p* > 0.95 were excluded from the figure for clarity. (** *p* < 0.01).

**Figure 4:**
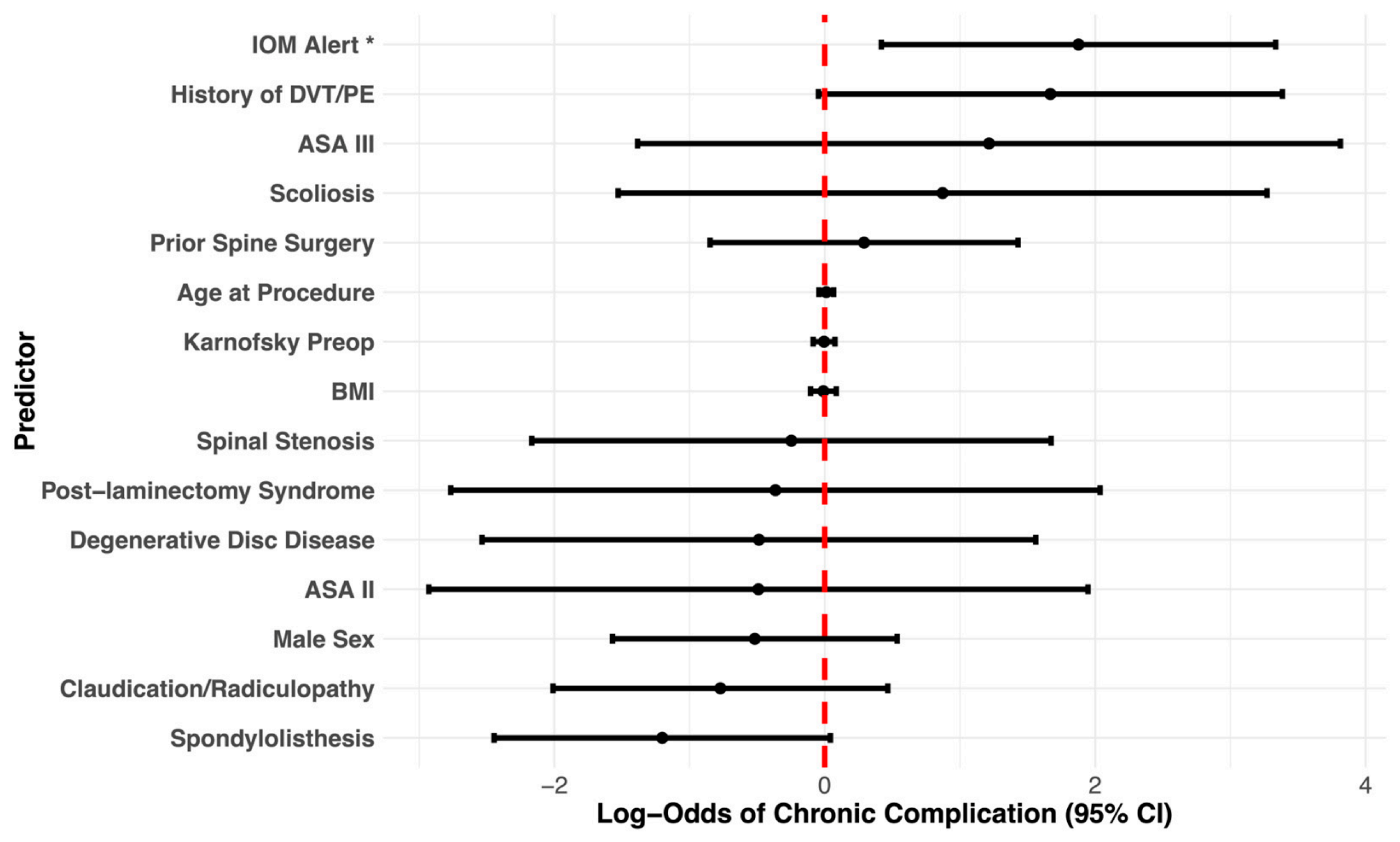
**Multivariate predictors of chronic complications.** Of the 22 variables included in our multivariate model, IOM alert was the only significant predictor of chronic postoperative complications, nearly doubling the log odds of a patient presenting with one or more of the following: postoperative foot drop, nerve root injury (excluding foot drop), thigh paresthesia, subsidence, or subsidence requiring revision. Predictors with *p* > 0.95 were excluded from the figure for clarity. (* *p* < 0.05).

Due to the small number of patients experiencing postoperative foot drop, a multivariate regression analysis of predictor variables was not feasible. When complications were pooled together into larger subgroups based shared characteristics (e.g., affected area, chronicity), the multivariate analysis demonstrated that an IOM alert was the only significant predictor of both new postoperative neurological deficits (*p* = 0.009) and chronic complications lasting longer than 6 months (*p* = 0.012) (Figure 3 and Figure 4). However, further sensitivity analyses excluding foot drop demonstrated no significant association between IOM alerts and either new postoperative neurological deficits or chronic complications. Given the low event rates (*n* = 7 and *n* = 27, respectively) and the inclusion of foot drop in both composite outcomes, these associations may be disproportionally driven by the foot drop signal and should therefore be interpreted cautiously (Figure 1).

All three patients who developed foot drop following an IOM alert demonstrated a decline in strength from 5/5 to at least 3/5 in the tibialis anterior, extensor hallucis longus, and/or gastrocnemius muscle(s). Each experienced significant intraoperative changes in SSEP recordings, while two of three also experienced MEP changes (Table 3). No patients experiencing foot drop had intraoperative EMG changes. One additional patient developed foot drop without an IOM alert; this patient had significant preoperative weakness in one extremity before developing contralateral foot drop following the procedure. Of the four total patients who experienced postoperative foot drop, two reported a full resolution within one year, whereas the remaining two continued to report persistent foot drop five years after their procedures. This variability in resolution was seen despite all four patients being engaged in routine physical and occupational therapy as part of their postoperative care within one year.

In addition to foot drop, the IOM alert was not significantly associated with any other intraoperative or postoperative complications. Similarly, the IOM alert was not associated with changes in the estimated blood loss, the length of hospital or ICU stay, or changes in the Karnofsky score. However, the IOM alert approached significance as a predictor of subsidence requiring revision and leukocytosis (both *p* = 0.06). No preoperative demographics, clinical variables, or surgical indications significantly predicted the occurrence of an IOM alert.

## 4. Discussion

### 4.1. MIS-TLIF and the Role of IOM

MIS-TLIF has emerged as a popular surgical technique for lumbar fusion due to its reduced tissue disruption, shorter recovery times, and lower infection rates compared to open procedures [14,15]. However, the minimally invasive nature of this technique also limits visualization and access, which may increase the risk of iatrogenic nerve injury. Intraoperative neuromonitoring may serve to mitigate these risks by providing near real-time electrophysiological feedback and clueing in the surgical team to potential postoperative complications while the procedure is ongoing. It has been argued that IOM is unwarranted due to higher costs and increased procedure times associated with its implementation [8,9,16]. Other studies have found that IOM use during spinal surgeries did not lead to significantly decreased postoperative neurological deficits [17,18], although this is debated [12]. Notably, Ament et al. [19] found an increase in the quality-of-life-years (QALYs) for patients undergoing spinal surgery with IOM as compared to those without.

In our study, we found that intraoperative neuromonitoring changes had a statistically significant association with postoperative foot drop. The high specificity and negative predictive value of IOM in our cohort suggest that it could potentially be used as an intraoperative screening tool to rule out iatrogenic injury resulting in foot drop. However, the low event rate of postoperative complications, including foot drop, in our cohort limited IOM’s predictive precision, as reflected by its low positive predictive value. These findings highlight important limitations in the use of IOM as a standalone screening tool in relatively low-risk populations and emphasize the need for the context-specific interpretation of alerts.

### 4.2. Limitations

Our study has several limitations. First, this retrospective study investigated a relatively small cohort (*n* = 439) at a single institution, which limits the generalizability of our findings. Additionally, the prevalence of IOM alerts in our cohort was low, occurring in only 4.8% of cases (21/439 patients). Several factors may contribute to this low rate within our analysis, including surgeon experience with MIS-TLIF and IOM usage at our institution.

Second, the low number of patients presenting with foot drop (*n* = 4) led to uncertainty in our multivariate model and limited its application for that particular complication [20]. To allow for multivariate analyses, outcomes were pooled into broader composite outcomes based on shared characteristics, such as neurological deficits and chronic complications. However, because these composite metrics included postoperative foot drop, the observed associations may reflect the influence of foot drop alone rather than demonstrating a broader predictive value of IOM. Further studies in larger cohorts are necessary to better define the clinical significance of these associations and overcome the statistical uncertainty inherent with low event rates.

Finally, although no preoperative or demographic variables were found to be associated with IOM alerts, our study did not evaluate the impact of several potential confounding factors, including the operative duration, surgeon experience, and prior surgical history. Additionally, because the study cohort spans an 11-year period, temporal changes in IOM technology, institutional practices, and surgeon-specific techniques may have occurred, which could impact results. Future studies controlling for these factors may help more clearly identify the independent relationship between IOM alerts and postoperative complications, especially foot drop, following MIS-TLIF.

## 5. Conclusions

Our study found IOM alerts during MIS-TLIF to be significantly associated with postoperative foot drop. The presence of an IOM alert was also associated with chronic postoperative complications and new neurological deficits, although the significance of these associations is less clear given that both composite outcomes included postoperative foot drop. IOM alerts demonstrated high specificity and negative predictive values for foot drop, although the rarity of this complication contributed to an overall low positive predictive value. Together, these findings suggest that IOM may be best utilized as an intraoperative screening tool to rule out iatrogenic foot drop. The lack of an IOM alert should therefore reassure surgeons that iatrogenic foot drop is unlikely, but presence of an alert should not be interpreted as definitive evidence that an injury is occurring. We hope future studies further assess the value of IOM as a screening tool in larger, higher-risk cohorts and investigate how IOM can influence postoperative care to reduce patient burdens and healthcare utilization and improve long-term outcomes.

## Figures and Tables

**Table 1:** Demographic data.

	No IOM Alert (*n* = 418)	IOM Alert (*n* = 21)	Overall (*n* = 439)
**Sex**			
Female	212 (50.7%)	10 (47.6%)	222 (50.6%)
Male	206 (49.3%)	11 (52.4%)	217 (49.4%)
**Age at Procedure**	61.0 [21.0, 90.0]	61.4 [28.0, 75.0]	61.0 [21.0, 90.0]
**BMI**	28.7 [17.1, 46.9]	27.8 [18.1, 41.2]	28.7 [17.1, 46.9]
**Smoking**			
No	279 (66.7%)	15 (71.4%)	294 (67.0%)
Yes	139 (33.3%)	6 (28.6%)	145 (33.0%)
**Drug Abuse**			
No	397 (95.0%)	20 (95.2%)	417 (95.0%)
Yes	21 (5.0%)	1 (4.8%)	22 (5.0%)
**Prior Spine Surgery**			
No	266 (63.6%)	17 (81.0%)	283 (64.5%)
Yes	152 (36.4%)	4 (19.0%)	156 (35.5%)
**# of Levels**			
1	357 (85.4%)	18 (85.7%)	375 (85.4%)
2	57 (13.6%)	3 (14.3%)	60 (13.7%)
3	4 (1.0%)	0 (0%)	4 (0.9%)
**Spinal Level**			
L4-L5	233 (55.7%)	12 (57.1%)	245 (55.8%)
L5-S1	103 (24.6%)	6 (28.6%)	109 (24.8%)
L4-S1	31 (7.4%)	3 (14.3%)	34 (7.7%)
L3-L5	20 (4.8%)	0 (0%)	20 (4.6%)
L3-L4	18 (4.3%)	0 (0%)	18 (4.1%)
L2-L3	6 (1.4%)	0 (0%)	6 (1.4%)
Other	7 (1.6%)	0 (0%)	7 (1.6%)

IOM, intraoperative monitoring; BMI, body mass index.

**Table 2:** Indications for minimally invasive transforaminal lumbar interbody fusion.

Surgical Indication	No IOM Alert (*n* = 418)	IOM Alert (*n* = 21)	Overall (*n* = 439)
Degenerative Disk Disease	322	14	4.35%
Stenosis	269	12	4.46%
Spondylolisthesis	261	12	4.60%
Claudication/Radiculopathy	182	10	5.49%
Scoliosis	27	1	3.70%
Synovial Cyst	25	1	4.00%

IOM, intraoperative monitoring.

**Table 3:** Classification of postoperative foot drop.

	IOM Alert Type	Muscle	Right Strength	Left Strength
		Tibialis Anterior	4/5	4/5
**Patient 1**	SSEP and MEP	Extensor Hallucis Longus	2/5	3/5
		Gastrocnemius	4/5	4/5
		Tibialis Anterior	4/5	4/5
**Patient 2**	SSEP and MEP	Extensor Hallucis Longus	4/5	3/5
		Gastrocnemius	4/5	4/5
		Tibialis Anterior	1/5	1/5
**Patient 3**	SSEP	Extensor Hallucis Longus	1/5	1/5
		Gastrocnemius	1/5	1/5
		Tibialis Anterior	1/5	2/5
**Patient 4**	None	Extensor Hallucis Longus	0/5	1/5
		Gastrocnemius	5/5	5/5

IOM, intraoperative monitoring; SSEP, somatosensory evoked potential; MEP, motor evoked potential. Preoperatively each patient was documented as full strength bilaterally, except for patient 4, who had previously demonstrated right-sided foot drop. Postoperatively, this remained unchanged. However, the patient developed new foot drop on the contralateral (left) side, which had previously been full strength.

## Data Availability

Deidentified data are available upon reasonable request.

## Abbreviations

The following abbreviations are used in this manuscript:

MIS-TLIF	Minimally invasive transforaminal lumbar interbody fusion
IOM	Intraoperative monitoring
MEP	Motor evoked potential
SSEP	Somatosensory evoked potential
EMG	Electromyography
TIVA	Total intravenous anesthesia
MAC	Minimum alveolar concentration
